# Global distribution patterns of siphonophores across horizontal and vertical oceanic gradients

**DOI:** 10.12688/openreseurope.18226.1

**Published:** 2024-08-13

**Authors:** Cristina Claver, Naiara Rodríguez-Ezpeleta, Xabier Irigoien, Oriol Canals

**Affiliations:** 1 Marine Research Division, Basque Research and Technology Alliance (BRTA), AZTI Foundation Sukarrieta, Sukarrieta, Vizcaya, 48395, Spain; 2 Education Research and Innovation Foundation, NEOM Base Camp, Building Number: 4758, Ocean Science and Solutions Applied Research Institute (OSSARI), NEOM, 49643, Saudi Arabia

**Keywords:** Siphonophores, DNA metabarcoding, gelatinous plankton, biogeography

## Abstract

**Background:**

Siphonophores are diverse, globally distributed hydrozoans that play a central role in marine trophic webs worldwide. However, they still constitute an understudied fraction of the open ocean gelatinous taxa, mainly due to challenges related to siphonophore sampling and identification, which have led to a general knowledge gap about their diversity, distribution and abundance.

**Methods:**

Here, we provide a global overview of the oceanic vertical distribution of siphonophores using DNA metabarcoding data from 77 bulk mesozooplankton samples collected at four different depth ranges (0-200, 200-500, 500-1000, 1000-3000 m depth) along the Atlantic, Pacific, and Indian Oceans during the MALASPINA-2010 circumnavigation expedition.

**Results:**

We detected a total of 44 siphonophore species (which represents about one quarter of the described siphonophore species) from which 26 corresponded to Calycophores, 14 to Physonectae and 2 to Cystonectae. Our results suggest wider horizontal and vertical distributions of siphonophore species than previously described, including novel records of some species in certain oceanic basins. Also, we provide insights into the intraspecific variation of widely distributed species. Finally, we show a vertical structuring of siphonophores along the water column; Calycophores (siphonophores without pneumatophores) dominated the epipelagic (from the surface to 200 m depth) and upper mesopelagic layers (from 200 to 500 m depth), while the proportion Physonectids (siphonophores with pneumatophore) notably increased below 500 meters and were dominant at bathypelagic depths (>1000 m depth).

**Conclusions:**

Our results support that the siphonophore community composition is vertically structured. Also, we provide insights into the potential existence of genetic variations within certain species that dominate some ocean basins or depth ranges. To our knowledge, this is the first time that DNA metabarcoding data is retrieved to study siphonophore distribution patterns, and the study provides evidence of the potential of molecular techniques to study the distribution of gelatinous organisms often destroyed in net sampling.

## Plain language summary

This study gives a worldwide view of where siphonophores live in the open ocean. To do so, we used genetic data from samples from different depths and ocean basins that were collected during a circumnavigation expedition. We identified 42 species, representing about a quarter of all known siphonophores. Some species were found in places they hadn’t been seen before so they seem to have wider distributions than previously thought. The study also looks at regional variations within species. Our results show that the siphonophore community is dominated by siphonophores without pneumatophores (gas-filled structure related with flotability) at shallow oceanic layers but dominated by siphonophores with pneumatophores in the deep sea. This is the first time that this kind of DNA data has been used to study the biogeography of these largely unknown creatures, showing it’s a useful method for studying organisms that are often damaged when collected with nets.

## Introduction

Siphonophores are marine worldwide distributed hydrozoans (Cnidaria) characterized by a complex colonial structure ( Mapstone, 2014). Although a few species float in the sea surface or are attached to the seafloor, most siphonophores inhabit the water column, from epipelagic to bathypelagic depths ( Mapstone, 2014). This is a morphologically diverse group classified in three different suborders, mainly differentiated by morphological features: Calycophorae have nectophores (swimming bell-like zooids for propulsion), Cystonectae have pneumatophores (gas-filled floats used to maintain orientation in the water and flotation) and Physonectae have both features ( Bidigare & Biggs, 1980).

Knowledge of siphonophore diversity has significantly improved recently due to advances in deep-sea exploration and increased scientific interest ( Hetherington *et al.*, 2022); to date, 190 species have been described ( WoRMS, 2023), the latest in 2023 ( Bolstad *et al.*, 2023). One of the reasons for the growing interest in understanding siphonophores is their unmeasured contribution to acoustic backscatter, since the pneumatophores of siphonophores and the swim bladders of teleost fish are targeted with the same frequencies and, thus, might be acoustically undistinguishable ( Barham, 1963; Barham, 1966; Kloser *et al.*, 2016; Warren *et al.*, 2001). Therefore, mesopelagic fish biomass estimates might be biased by siphonophores ( Proud *et al.*, 2019). Despite these efforts, and as for most gelatinous organisms, siphonophores still constitute an understudied fraction of the deep-sea gelatinous zooplankton and yet they represent up to 25% of the total pelagic biomass with and important role in trophic webs ( Hetherington *et al.*, 2022; Robison, 2004).

As such, most of the research on siphonophores has been performed at regional scales, has considered a few of the described species, and has not deepen into the vertical distribution of some groups, which could be key to understand the different trophic niches they occupy in pelagic ecosystems ( Hetherington *et al.*, 2022). Knowledge gaps in siphonophore diversity, distribution and abundance can be attributed to the difficulty in sampling and identifying them. Siphonophores are fragile and easily broken when caught with nets, meaning that caught individuals are often damaged and almost impossible to classify to species or genus level by visual methods (e.g. ( Fernández de Puelles *et al.*, 2019)). The use of in situ video has contributed to understand distribution and abundance but does not provide accurate taxonomy ( Robison *et al.*, 1998).

In this context, the combination of oceanographic expeditions with molecular taxonomic approaches could be key to study siphonophore diversity, abundance and diet ( Damian-Serrano *et al.*, 2022; Govindarajan *et al.*, 2021). Indeed, oceanographic campaigns gather massive sample collections and datasets that usually are underutilized. Considering the cost of these surveys, unprocessed data use or cross-disciplinary reanalysis is a source of potentially relevant information given different research perspectives and promotes outcome maximization, as well as the best use of resources ( Kennicutt *et al.*, 2016). Bulk samples and, in recent years, environmental samples (water or sediment) are among the most collected material in research vessels. Regardless of the specific purpose for which these samples are collected, these samples can be analyzed through DNA metabarcoding, a technique that has demonstrated its utility for siphonophore detection, although most of the studies have aimed to characterize the general planktonic community ( Bucklin *et al.*, 2010; Di Capua *et al.*, 2021; Govindarajan *et al.*, 2021; Parry *et al.*, 2021). To our knowledge, a single study has applied this technique to siphonophores, in particular, to the study of their diets ( Damian-Serrano *et al.*, 2022). Yet, although little used, this technique has the potential to fill knowledge gaps about diversity, distribution and abundance of these organisms at a global scale.

Here, we have used siphonophore molecular data from samples collected during the MALASPINA-2010 circumnavigation expedition to increase knowledge about siphonophore horizontal and vertical distribution patterns along the global ocean. For some species, this study provides new records and suggests wider horizontal and vertical distributions than previously described. Also, we provide insights into the intraspecific variation of widely distributed siphonophores.

## Methods

### Siphonophore datasets compilation

**Figure 1.**
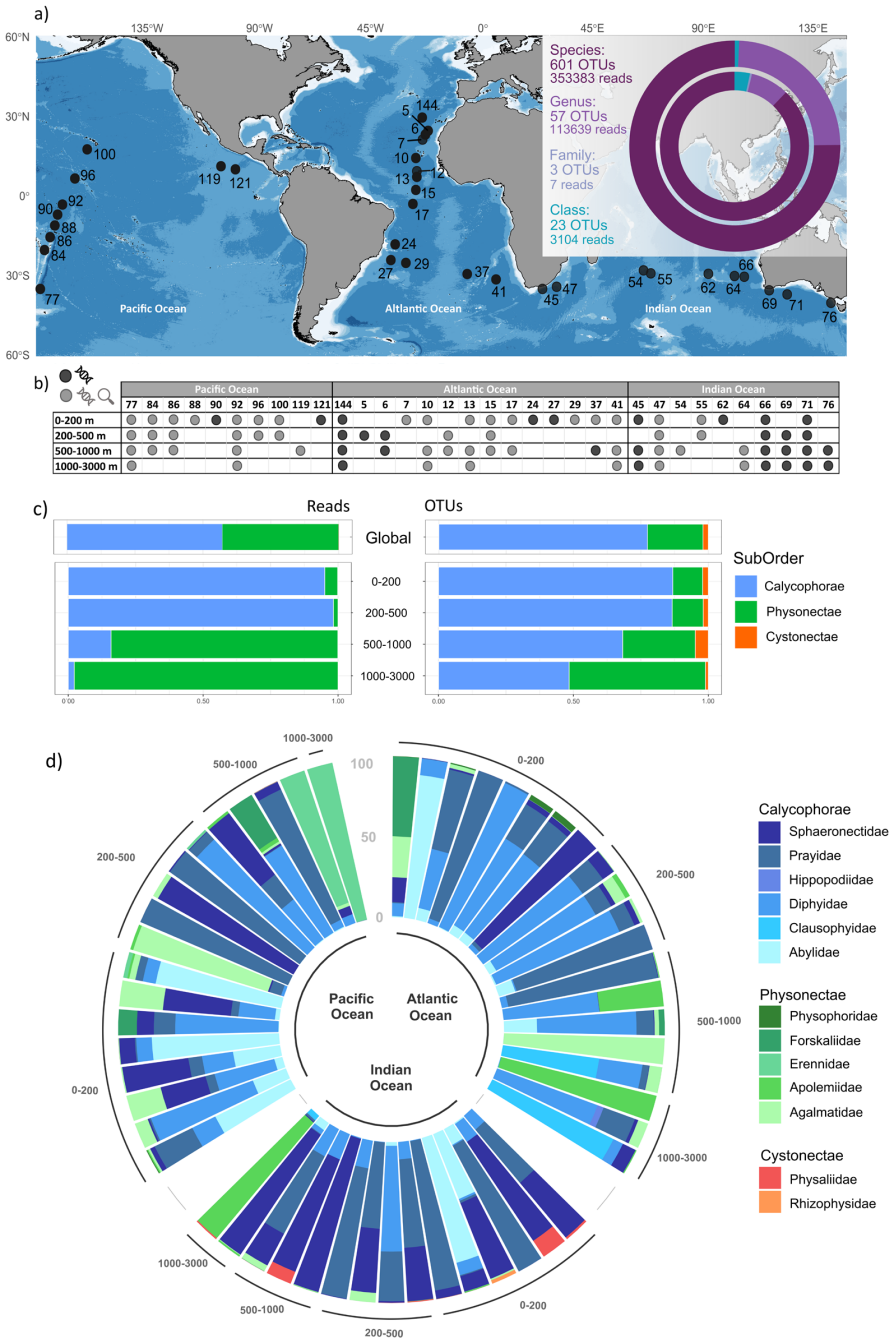
Global overview of siphonophore diversity and distribution. **a)** Location of the sampling stations and taxonomic depth obtained for siphonophores, showing the proportion of reads (outer doughnut) and OTUs (inner doughnut) assigned to class, family, genus, and species level. **b)** Depth ranges from where samples were analyzed in this study. Black dots represent samples with only DNA data and grey dot with both DNA and morphological data. **c)** Global relative read abundance (left) and proportion of OTUs (right) of siphonophore suborders by depth ranges. **d)** Relative read abundance plot representing distribution of siphonophore families by depth ranges. Only samples with more than 100 siphonophore reads are represented.

Data used in the present study was extracted from the molecular dataset compiled in Canals *et al.* (2024), where details on the sample processing, DNA extraction, library preparation, and taxonomic assignment are provided. Canals *et al.* analyzed 77 bulk zooplankton samples collected during the Malaspina circumnavigation expedition ( Duarte, 2015) ( Figure 1a, b) covering 34 different stations across the Atlantic, Indian, and Pacific oceans, from the surface down to 3000 m depth. For this study, the *mlCOIint* (313 bp-long region from the cytochrome oxydase I gene; Leray *et al.* (2013)) sequences assigned to Siphonophorae (Cnidaria, Hydrozoa) were selected. Although metabarcoding data for another marker (mac18S from the 18S rRNA gene) was also available from ( Canals *et al.*, 2024), only *mlCOIint* was considered due to its higher potential to classify sequences at the species level and to detect intraspecific variability ( Turon *et al.*, 2020).

### Diversity descriptors and statistical analysis

All statistical analyses were performed in R environment 4.2.2 ( www.r-project.org). Alpha-diversity analysis was based on the richness (number of taxa detected in each sample) and Shannon diversity index (calculated using the diversity function, vegan v2.6-4 R package) ( Oksanen *et al.*, 2022) on samples rarefied to 500 reads ( *rrarefy* function, vegan R package) to avoid biases due to different sequencing effort. Beta-diversity analysis was based on Bray-Curtis dissimilarity between pairs of samples ( *vegdist* function, *vegan* R package). The proportion of dissimilarity attributed to balanced variation in species abundances (equivalent to turnover for incidence-based indices) and abundance gradient (equivalent to nestedness) ( Baselga, 2013) was assessed using the *beta.pair.abund* function of the *betapart* R package (version 1.6; ( Baselga & Orme, 2012)). Ordination analysis was performed by nonmetric multidimensional scaling (NMDS; metaMDS function, vegan R package) on log transformed data, and ANOSIM test ( *anosim* function, vegan R package) was applied to test the significance of ordination of communities according to predefined groups.

## Results

### Overview of siphonophore diversity

Overall, 470,133 siphonophore reads (4,5% of total metazoan reads) were retrieved. These reads were clustered into 666 Operational Taxonomic Units (OTUs) from which 88% were taxonomically assigned to species level, 8.5% to genus, 0.5 to family, and about 3% remained as Siphonophorae ( Figure 1a). Calycophorae appeared as the most abundant and diverse suborder (26 species representing 64% of the siphonophore reads), followed by Physonectae (14 species, 35% of reads) and Cystonectae (only 2 species and <1% of reads). Some species such as *Nectadamas diomedeae* or *Sphaeronectes koellikeri* had a high number of OTUs assigned (>65 OTUs) whereas other species had a single OTU, such as *Lensia exeter*.

### Distribution patterns

Siphonophores were detected at all oceanic basins and depth ranges under study. Calycophores, represented by Abylidae and Diphydae families, were clearly dominant in epipelagic (from surface to 200 m depth) and upper mesopelagic (from 200 to 500 m depth) layers. Yet, a sharp increase in Physonectae reads was detected below 500 m ( Figure 1c), including species in the Apolemidae and Erennidae families. Cystonectae displayed low read abundances along the whole water column, mainly located in the Indian Ocean. Although some species were found in all the water column, they were mainly present in a certain depth range (refer to extended data: Figure S1; Claver *et al.*, 2024).

**Figure 2.**
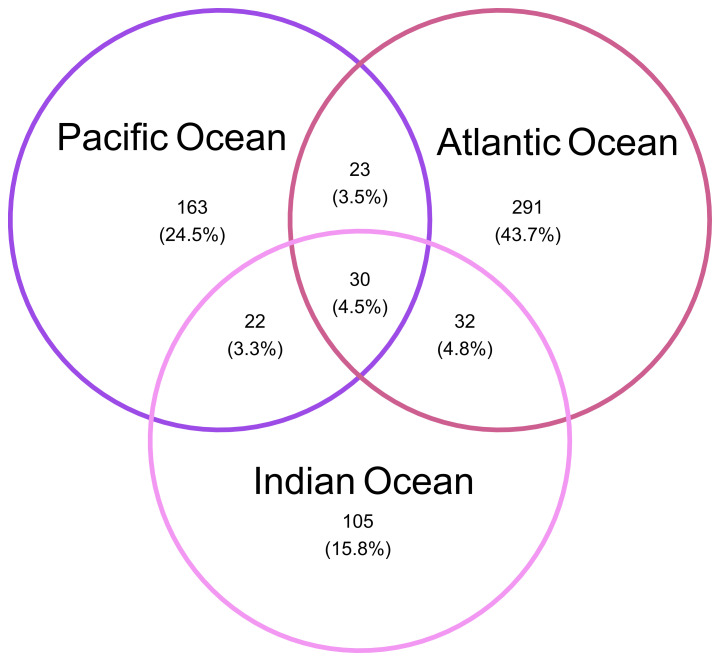
Venn diagram representing the siphonophore OTUs that are detected in the three ocean basins.

**Figure 3.**
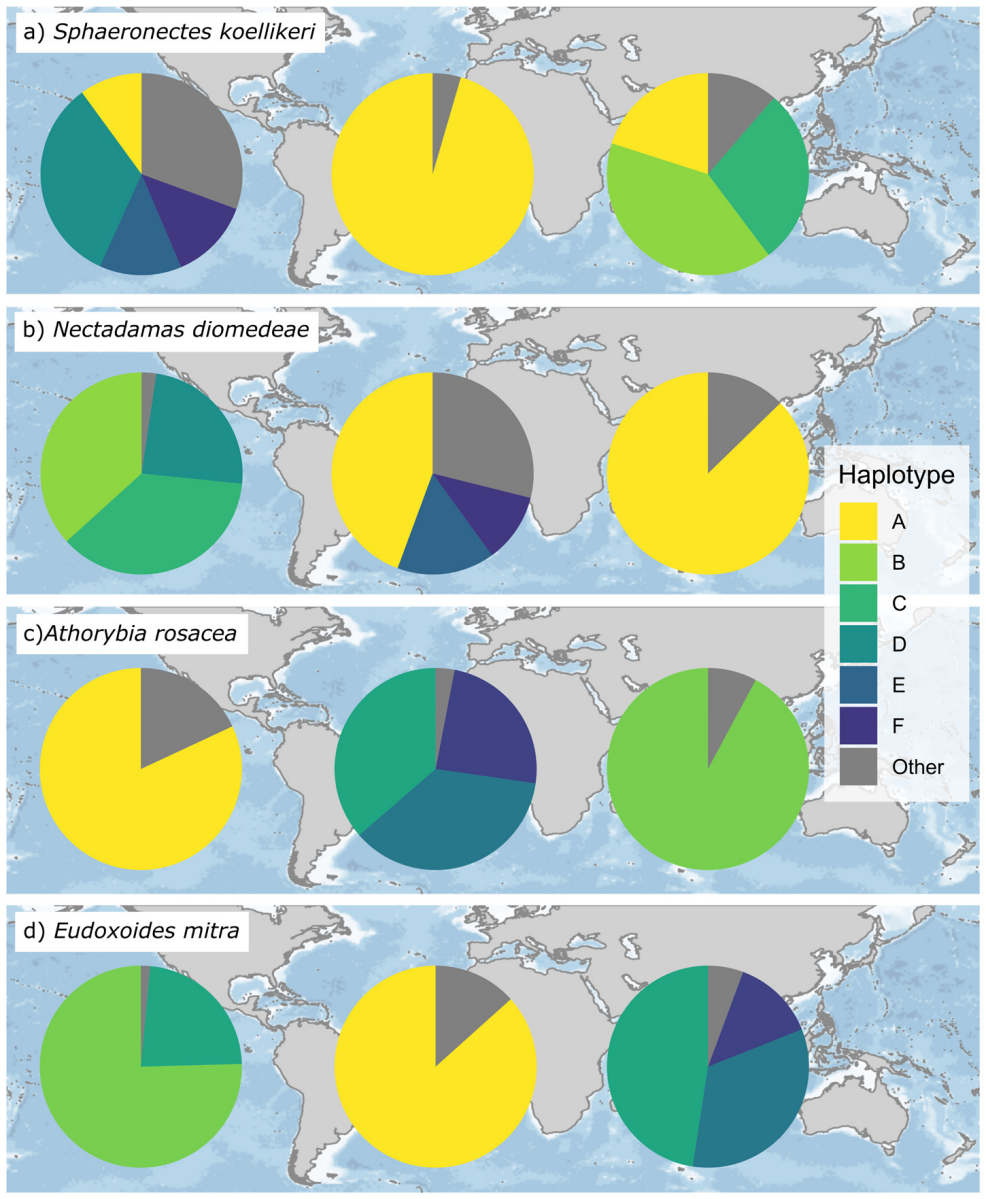
Intraspecific variability of four siphonophore species across the Pacific, Atlantic and Indian Oceans. The pie graphs represent the relative abundance of different haplotypes in each ocean basin. The grey section of the pies comprises haplotypes representing less than 10% of the abundance in that basin.

In the horizontal gradient, both proportion of reads and OTUs corresponding to Cystonectae were small globally (0.01% and 2%, respectively) ( Figure 1c). Within Calycophores, Prayidae and Sphaeronectidae were the most distributed families in all three ocean basins. Among Cystonectae, the Physaliidae family was primarily detected in the Indian Ocean ( Figure 1d). The Atlantic Ocean was the ocean basin with the highest number of assigned species and OTUs followed by the Pacific and the Indian Ocean ( Table 1). About half of the species were present in all ocean basins, 12 species were shared only by two ocean basins and 10 were exclusively found in the Atlantic or Pacific Ocean (refer to extended data: Figure S2; Claver *et al.*, 2024). Although most of the species were shared by the different ocean basins 85% of the OTUs were found exclusively in a single ocean basin, being the Pacific Ocean the ocean basin with highest number of OTUs not classified to species level (36 OTUs), followed by the Atlantic (18 OTUs) and the Indian Ocean (14 OTUs) ( Figure 2). For some widely distributed species with high intraspecific variation (>10 OTUs) we identified biogeographical patterns and basin-exclusive OTUs ( Figure 3).

**Table 1.** Taxonomic assignments, represented percentage and number of siphonophore OTUs found in each ocean basin.

SubOrder	Family	Genus	Species	OTUs (%)	Pacific Ocean	Atlantic Ocean	Indian Ocean
Calycophorae	Abylidae	Abylopsis	*Abylopsis eschscholtzii*	4.1	19	6	12
Calycophorae	Abylidae	Abylopsis	*Abylopsis tetragona*	0.4	2	1	1
Calycophorae	Abylidae	Bassia	*Bassia bassensis*	0.9	3	3	3
Calycophorae	Abylidae	Ceratocymba	*Ceratocymba * *sagittata*	0.4	1	1	1
Calycophorae	Clausophyidae	Chuniphyes	*Chuniphyes * *multidentata*	0.9		6	3
Calycophorae	Diphyidae	Dimophyes	*Dimophyes arctica*	0.1			1
Calycophorae	Diphyidae	Diphyes	*Diphyes bojani*	8.8	3	57	1
Calycophorae	Diphyidae	Diphyes	*Diphyes dispar*	6.4	6	38	3
Calycophorae	Diphyidae	Diphyes	unclassified	2.2	14	2	
Calycophorae	Diphyidae	Eudoxoides	*Eudoxoides mitra*	4.1	12	12	13
Calycophorae	Diphyidae	Eudoxoides	*Eudoxoides spiralis*	1.8		12	3
Calycophorae	Diphyidae	Lensia	*Lensia achilles*	0.6	3	3	1
Calycophorae	Diphyidae	Lensia	*Lensia campanella*	6.9	22	26	4
Calycophorae	Diphyidae	Lensia	*Lensia conoidea*	0.6	2	2	2
Calycophorae	Diphyidae	Lensia	*Lensia exeter*	0.1		1	1
Calycophorae	Diphyidae	Lensia	*Lensia fowleri*	1.2	5	4	4
Calycophorae	Diphyidae	Lensia	*Lensia hotspur*	1.5	4	5	3
Calycophorae	Diphyidae	Lensia	*Lensia multicristata*	0.6	1	2	1
Calycophorae	Diphyidae	Lensia	unclassified	1.8	6	4	6
Calycophorae	Diphyidae	Sulculeolaria	*Sulculeolaria * *quadrivalvis*	0.1	1	1	
Calycophorae	Diphyidae	unclassified	unclassified	0.4	3		
Calycophorae	Hippopodiidae	Hippopodius	*Hippopodius * *hippopus*	0.1		1	
Calycophorae	Hippopodiidae	Vogtia	*Vogtia spinosa*	0.1	1		
Calycophorae	Prayidae	Amphicaryon	*Amphicaryon acaule*	1.2	5	2	2
Calycophorae	Prayidae	Amphicaryon	unclassified	0.4	1	1	2
Calycophorae	Prayidae	Lilyopsis	*Lilyopsis medusa*	0.3	2		
Calycophorae	Prayidae	Nectadamas	*Nectadamas * *diomedeae*	18.9	29	66	52
Calycophorae	Prayidae	Praya	*Praya reticulata*	0.1	1		
Calycophorae	Prayidae	Rosacea	*Rosacea flaccida*	0.1	1		1
Calycophorae	Sphaeronectidae	Sphaeronectes	*Sphaeronectes * *koellikeri*	9.6	36	29	31
Physonectae	Agalmatidae	Agalma	*Agalma elegans*	1.0	6	2	
Physonectae	Agalmatidae	Athorybia	*Athorybia rosacea*	1.9	7	4	4
Physonectae	Agalmatidae	Frillagalma	*Frillagalma vityazi*	0.3		2	
Physonectae	Agalmatidae	Halistemma	*Halistemma cupulifera*	0.1		1	
Physonectae	Agalmatidae	Halistemma	*Halistemma rubrum*	1.0	7	1	
Physonectae	Agalmatidae	Halistemma	unclassified	1.3	1	9	1
Physonectae	Agalmatidae	Marrus	*Marrus claudanielis*	0.1	1		
Physonectae	Agalmatidae	Marrus	unclassified	0.7	1	2	2
Physonectae	Agalmatidae	Nanomia	*Nanomia bijuga*	1.3	3	4	1
Physonectae	Apolemiidae	Apolemia	*Apolemia lanosa*	5.7	4	29	2
Physonectae	Apolemiidae	Apolemia	*Apolemia rubriversa*	0.1		1	
Physonectae	Apolemiidae	Apolemia	unclassified	1.8	2	1	4
Physonectae	Erennidae	Erenna	*Erenna cornuta*	0.3		2	
Physonectae	Erennidae	Erenna	*Erenna laciniata*	1.0	7	1	1
Physonectae	Erennidae	Erenna	unclassified	0.1	1		
Physonectae	Forskaliidae	Forskalia	*Forskalia asymmetrica*	0.3	1	2	
Physonectae	Forskaliidae	Forskalia	*Forskalia tholoides*	1.6	1	7	
Physonectae	Physophoridae	Physophora	*Physophora * *hydrostatica*	1.0	2	3	2
Cystonectae	Physaliidae	Physalia	*Physalia physalis*	1.3	1	1	9
Cystonectae	Rhizophysidae	Rhizophysa	*Rhizophysa filiformis*	0.6	2		2
unclassified	unclassified	unclassified	unclassified	3.4	8	1	1
		**Total**	**Species** **OTUs**	**42** **666**	**34** **238**	**36** **358**	**24** **180**

### Alpha- and beta-diversity patterns of siphonophores

**Figure 4.**
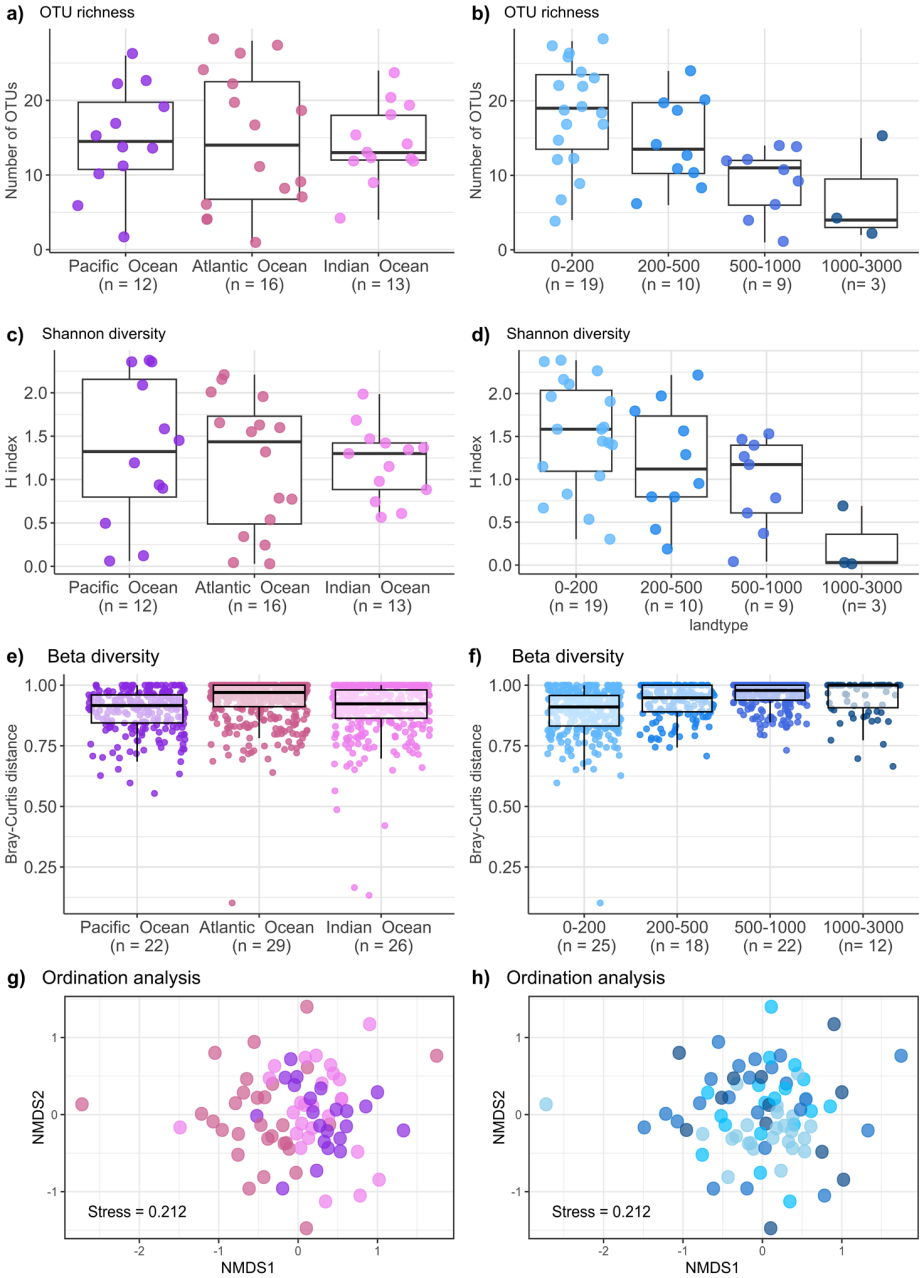
Alpha- and beta-diversity analyses of Siphonophores. Samples are grouped by ocean basin (left) or by depth (right), indicating the number of samples included in each category (n). Boxplots show different measurements: OUT richness ( **a,b**), Shannon diversity ( **c,d**) and Bray-Curtis distances ( **e,f**). Non-metric multidimensional scaling (NMDS) analysis of siphonophores based on Bray-Curtis distances, colored **f)** by ocean (ANOSIM R= 0.117, p = 0.0001) and h) depth (ANOSIM R = 0.021, p = 0.45).

Alpha-diversity measurements did not show horizontal patterns across all the three ocean basins because siphonophore communities showed similar number of OTUs ( Figure 4a; refer to extended data: Figure S3a; Claver *et al.*, 2024). However, a progressive decreasing pattern in siphonophore richness was observed with depth, with the highest number of OTUs occurring in the epipelagic zone ( Figure 4b; refer to extended data: Figure S3b; Claver *et al.*, 2024). Accordingly, all ocean basins showed similar diversity values ( Figure 4c; refer to extended data: Figure S3c; Claver *et al.*, 2024) whereas epipelagic samples showed the highest diversity among depth ranges ( Figure 4d; refer to extended data: Figure S3d; Claver *et al.*, 2024). Beta-diversity patterns were not consistent across oceans because samples by ocean basin presented low dissimilarities ( Figure 4e; refer to extended data: Figure S3e,g; Claver *et al.*, 2024), whereas a vertical pattern was more evident and epipelagic samples showed smallest dissimilarities between them than the bathypelagic samples ( Figure 4f; refer to extended data: Figure S3f,h; Claver *et al.*, 2024). The components of the dissimilarity were broken down and the proportion of dissimilarity attributed to balanced variation in species abundances (equivalent to turnover) resulted to be the major component, with some minor proportion of abundance gradient (equivalent to nestedness) in the epipelagic and upper mesopelagic layers (refer to extended data: Figure S4; Claver *et al.*, 2024). The ordination analysis of the communities weakly supported a horizontal structuring based on ocean basin ( Figure 4g), whereas the vertical structuring was statistically supported to be the main factor determining the siphonophore community ( Figure 4h). These patterns became more evident when vertical samples where split into ocean basin and *vice versa* (refer to extended data: Figure S5; Claver *et al.*, 2024).

## Discussion

### Filling knowledge gaps in siphonophore ecology

Siphonophores constitute an understudied taxa of the gelatinous plankton, and their role in oceanic biogeochemical processes is barely understood due to lack of global estimates of horizontal and vertical distribution patterning ( Hetherington *et al.*, 2022). The study of siphonophores at community level has recently been highlighted to become a priority in view of their correlations with hydrographical features (such as water temperature and salinity) and the changes that the oceans are undergoing ( Park *et al.*, 2023).

To our knowledge, this is the most extensive original work on siphonophores and one of the studies that describes more diversity. Here, we provide insights into global diversity and distribution of siphonophores across the water column in tropical and subtropical regions. For some species, this study provides new records and suggests wider horizontal and vertical distributions than previously described. Also, the occurrence of certain siphonophore species in this study surpasses the number of records gathered in the last century (see Extended data Figure S2; Claver *et al.*, 2024). Our results indicate that the siphonophore community composition is vertically structured, which is in line with previous knowledge ( Mapstone, 2014). The decrease of the alpha-diversity with depth is consistent with whole mesozooplankton patterns ( Canals *et al.*, 2024), although its decrease through the mesopelagic zone is minimal and suggests a high diversity in the mesopelagic layers, where most of the species are found ( Mapstone, 2014). The increase of the beta-diversity with depth indicates that the highest dissimilarities in siphonophore communities are found in the deepest layers, where low connectivity for mesozooplankton communities has already been found ( Canals *et al.*, 2024).

Although most of the siphonophores are cosmopolitan ( Mapstone, 2014), preferred latitudinal ranges have been described for some species ( Mackie *et al.*, 1988) as well as allopatric relations within genus ( Uribe-Palomino *et al.*, 2019). Thus, the sampling area has potentially limited the coverage of species diversity because most of the sampling points included in this study are in tropical and subtropical regions (35 ºN – 40 ºS), resulting in a broad but not complete detection of siphonophore diversity. Species that have not been detected in this study may be more abundant in other ranges such as temperate zones or polar regions. Also, sampling points in the Indian Ocean cover a significantly narrow latitudinal range compared to the other two ocean basins. This could explain the lower number of species and OTUs identified in that basin.

### Future siphonophore research requires methodological advances

Molecular approaches have revolutionized marine ecosystem monitoring in the last decades by providing a cost-effective additional tool ( Danovaro *et al.*, 2016; Suter *et al.*, 2021). Specially, bulk DNA metabarcoding has identified more species than morphological methods and provided higher taxonomical resolution for hydrozoans ( Deagle *et al.*, 2018). This is the first time that DNA metabarcoding data is retrieved to study siphonophore distribution patterns, identifying more than 20 percent of the siphonophore species described in the world, some of which have been found in previously unrecorded ocean basins.

Referring to an elusive and understudied taxa such as siphonophores, the application of genetics can also provide exclusive valuable information ( Schwartz *et al.*, 2007). For instance, the choice of a region of the COI gene has allowed to determine intraspecific variability within the identified species, information not obtainable with morphological analyses. We have identified several species with a high number of OTUs, such as *Nectadamas diomedeae* or *Sphaeronectes koellikeri*, among others. Although these species are found in all three basins, some of the most abundant OTUs are exclusively found in one ocean, suggesting that they might correspond to regional variations ( Turon *et al.*, 2020). Interestingly, although the Indian Ocean does not include any species unique to that area, more than half of the OTUs in the Indian Ocean are unique to that basin (see Figure 3) which supports the existence of phylogeographic patterns derived from evolutionary adaptations. Moreover, a vertical OTU distribution across the water column was identified for some siphonophore species such as *Eudoxoides mitra* and *Nectadamas diomedeae* (see extended data Figure S6; Claver *et al.*, 2024). Indeed, intraspecific variability has been shown to have a positive effect in the ecology of some marine invertebrates ( Gamfeldt *et al.*, 2005; Jacobs & Podolsky, 2010), and may also be relevant for these taxa. The assignment of multiple OTUs to the same species can also be explained by the existence of hidden diversity ( Etter *et al.*, 1999) or due to lack of completeness of the genetic reference databases ( Claver *et al.*, 2023). Other authors have identified cryptic diversity among some of the siphonophores detected in this study, such as *Physalia* genus ( Pontin & Cruickshank, 2012), for which we found 9 different OTUs. Finally, some of the very low-abundant OTUs might not represent real biological variability. In our data, the species with most OTUs assigned are also the ones with the highest number of reads. While it is logical to observe greater variability in larger populations, it could also be the case that abundant sequences accumulate more sequencing errors and chimeric sequences. Still, biogeographic patterns derived from the most abundant OTUs are expected to represent biologically meaningful variability.

A benefit from metabarcoding studies is that sequencing data can be re-analyzed unlimitedly. Even if the target taxa of the original study are different, information about siphonophores can be retrieved from broad-range markers as described in this study. Initiatives such as the Tara Oceans project ( Sunagawa *et al.*, 2020), in which molecular data is publicly shared and openly accessible, represent a valuable source of information that can revolutionize the field of siphonophore research in the coming years. In our case, sampling characteristics of the original study were not fit for siphonophores but for mesozooplankton in general (see Canals *et al.*, 2024) which limits the detection of certain siphonophore species. Because sampling was carried out in the water column with plankton nets, neustonic and benthic siphonophores are much likely underrepresented; indeed, no benthic siphonophore species was detected. However, within Cystonectae, which are epipelagic or neustonic siphonophores, two species ( *Physalia physalis* and *Rhizophysa filiformis*) were detected in more than two ocean basins ( Table 1; refer to extended data Figure S2; Claver *et al.*, 2024) across the water column. Although adult individuals float, early stages develop in deep waters ( Munro *et al.*, 2019), meaning that it is likely that detections below the epipelagic layer correspond to eggs or larvae. Also, a prefiltering mesh was used to collect the planktonic samples, which might avoid capturing large siphonophores. Still, fragments of big siphonophores can enter the net when breaking the colonies. Here, we have identified species such as *Apolemia lanosa*, which lengths 2 meters ( WoRMS, 2023). So, considering that it is an opportunistic analysis, the effectiveness is quite reasonable.

Our results are consistent with previous studies that, for these fragile taxa, molecular tools may outperform traditional methodologies ( Govindarajan *et al.*, 2021). However, the potential of metabarcoding assessment can be limited by the completeness and accuracy of genetic refence databases used for the taxonomic assessment ( Claver *et al.*, 2023). To date, more than half of the described siphonophore species have available reference sequences in GenBank (64 out of 110 Calycophorae, 38 out of 75 Physonectae and 4 out of 5 Cystonectae). For an understudied group, 56% of completeness is a high value, considering that reference databases of other more extensively studied taxa have similar coverages ( Weigand *et al.*, 2019). Overall, Calycophorae had less unclassified reads (5%) than Physonectae (26%), which suggest that the reference database is most likely incomplete for the latter. Indeed, Physonectids are the most abundant siphonophores below the mid mesopelagic (>500 m), depths reported to be the most unknown by other authors ( Canals *et al.*, 2024; Sommer *et al.*, 2017). Interestingly, although for some genus all species were represented in the database, we detected OTUs assigned to the genus, suggesting certain level of hidden diversity. This is the case of *Amphicaryon* genus, for which all the described species have reference sequences but 3 OTUs are assigned to *Amphicaryon sp*, being 2 of the OTUs exclusive from one ocean basin. It is worth mentioning that effort is being made for improving genetic resources for siphonophore research. For instance, reference libraries for taxonomic assignments are being completed by barcoding new species ( Ortman *et al.*, 2010) and metabarcoding markers that amplify siphonophores are being developed ( Jarman *et al.*, 2013). These advances are making it possible to carry out genetic studies such as the one described here.

Since metabarcoding does not provide absolute values, compositional data only allows to compare variations in siphonophore proportion among different depths, which represents the importance of a certain group across the water column. These limitations in terms of abundance or biomass estimates preclude the direct use to determine how much siphonophores contribute to acoustic uncertainty in fish biomass estimations, which is partly attributed to the presence of siphonophores with pneumatophore (gas-filled structure also giving acoustic signal) from the suborders Physonectae and Cystonectae ( Proud *et al.*, 2019). However, a combination of simple estimates of abundance (image analysis) or biomass (wet weight) with metabarcoding approaches could result in an effective way to study the distribution of different taxonomic groups at global scales.

## Data Availability

Raw sequence data and associated metadata are available on the NCBI SRA (BioProject PRJNA1033987). *https://www.ncbi.nlm.nih.gov/bioproject/PRJNA1033987* ( Canals *et al.*, 2024) Additional material is publicly available in Zenodo under the repository entitled: Data from: Global distribution of siphonophores across horizontal and vertical oceanic gradients https://doi.org/10.5281/zenodo.12720803 ( Claver *et al.*, 2024) This repository contains the following supplementary data: Figure S1. Vertical distribution of siphonophore species identified in this study. Figure S2. Global distribution of siphonophore species identified in this study. Figure S3. Alpha-diversity and beta-diversity measurements of Siphonophorae by ocean basin and by depth. Figure S4. Breakdown of beta-diversity by depth. Figure S5. Breakdown of ordination analysis by ocean basin for epipelagic, upper mesopelagic, low mesopelagic and bathypelagic samples. Figure S6. Horizontal and vertical interspecific variability found in two siphonophore species. Data are available under the terms of the Creative Commons Attribution 4.0 International license (CC-BY 4.0).

## References

[ref-1] Barham EG : Siphonophores and the deep scattering layer. *Science.* 1963;140(3568):826–828. 10.1126/science.140.3568.826 17746436

[ref-2] Barham EG : Deep scattering layer migration and composition: observations from a diving saucer. *Science.* 1966;151(3716):1399–1403. 10.1126/science.151.3716.1399 17817303

[ref-3] Baselga A : Separating the two components of abundance-based dissimilarity: balanced changes in abundance vs. abundance gradients. *Methods Ecol Evol.* 2013;4(6):552–557. Ecography, 36, 124-128. 10.1111/2041-210X.12029

[ref-4] Baselga A Orme CDL : *betapart*: an R package for the study of beta diversity. *Methods Ecol Evol.* 2012;3(5):808–812. 10.1111/j.2041-210X.2012.00224.x

[ref-5] Bidigare RR Biggs DC : The role of sulfate exclusion in buoyancy maintenance by siphonophores and other oceanic gelatinous zooplankton. *Comp Biochem Physiol A Physiol.* 1980;66(3):467–471. 10.1016/0300-9629(80)90193-0

[ref-6] Bolstad K Amsler M Broyer CD : In-situ observations of an intact natural whale fall in Palmer deep, Western Antarctic Peninsula. *Polar Biol.* 2023;46(2):123–132. 10.1007/s00300-022-03109-1

[ref-7] Bucklin A Ortman BD Jennings RM : A “Rosetta Stone” for metazoan zooplankton: DNA barcode analysis of species diversity of the Sargasso Sea (Northwest Atlantic Ocean). *Deep Sea Res 2 Top Stud Oceanogr.* 2010;57(24–26):2234–2247. 10.1016/j.dsr2.2010.09.025

[ref-8] Canals O Corell J Villarino E : Global mesozooplankton communities show lower connectivity in deep oceanic layers.2024; e17286. 10.1111/mec.17286 38287749

[ref-9] Claver C Canals O de Amézaga LG : An automated workflow to assess completeness and curate GenBank for environmental DNA metabarcoding: the marine fish assemblage as case study. *Environmental DNA.* 2023;5(4):634–647. 10.1002/edn3.433

[ref-10] Claver C Rodriguez-Ezpeleta N Irigoien X : Data from: global distribution of siphonophores across horizontal and vertical oceanic gradients. [Data set]. En Open Research Europe. Zenodo.2024. 10.5281/zenodo.12720803 PMC11399771 39279823

[ref-11] Damian-Serrano A Hetherington ED Choy CA : Characterizing the secret diets of siphonophores (Cnidaria: Hydrozoa) using DNA metabarcoding. *PLoS One.* 2022;17(5): e0267761. 10.1371/journal.pone.0267761 35594271 PMC9122208

[ref-12] Danovaro R Carugati L Berzano M : Implementing and innovating marine monitoring approaches for assessing marine environmental status. *Front Mar Sci.* 2016;3:213. 10.3389/fmars.2016.00213

[ref-13] Deagle BE Clarke LJ Kitchener JA : Genetic monitoring of open ocean biodiversity: an evaluation of DNA metabarcoding for processing Continuous Plankton Recorder samples. *Mol Ecol Resour.* 2018;18(3):391–406. 10.1111/1755-0998.12740 29171158

[ref-14] Di Capua I Piredda R Mazzocchi MG : Metazoan diversity and seasonality through eDNA metabarcoding at a Mediterranean Long-Term Ecological Research site. *ICES J Mar Sci.* 2021;78(9):3303–3316. 10.1093/icesjms/fsab059

[ref-15] Duarte CM : Seafaring in the 21st century: the Malaspina 2010 circumnavigation expedition.2015;24(1):11–14. 10.1002/lob.10008

[ref-16] Etter RJ Rex MA Chase MC : A genetic dimension to deep-sea biodiversity. *Deep Sea Research Part I: Oceanographic Research Papers.* 1999;46(6):1095–1099. 10.1016/S0967-0637(98)00100-9

[ref-17] Fernández de Puelles ML Gazá M Cabanellas-Reboredo M : Zooplankton abundance and diversity in the tropical and subtropical ocean. *Diversity.* 2019;11(11):203. 10.3390/d11110203

[ref-18] Gamfeldt L Wallén J Jonsson PR : Increasing intraspecific diversity enhances settling success in a marine invertebrate. *Ecology.* 2005;86(12):3219–3224. 10.1890/05-0377

[ref-19] Govindarajan AF Francolini RD Jech JM : Exploring the use of environmental DNA (eDNA) to detect animal taxa in the mesopelagic zone. *Front Ecol Evol.* 2021;9: 574877. 10.3389/fevo.2021.574877

[ref-20] Hetherington ED Damian-Serrano A Haddock SHD : Integrating siphonophores into marine food-web ecology. *Limnol Oceanogr Lett.* 2022;7(2):81–95. 10.1002/lol2.10235

[ref-21] Jacobs MW Podolsky RD : Variety is the spice of life histories: comparison of intraspecific variability in marine invertebrates. *Integr Comp Biol.* 2010;50(4):630–642. 10.1093/icb/icq091 21558229

[ref-22] Jarman SN McInnes JC Faux C : Adélie penguin population diet monitoring by analysis of food DNA in scats. *PLoS One.* 2013;8(12): e82227. 10.1371/journal.pone.0082227 24358158 PMC3864945

[ref-23] Kennicutt MC Kim YD Rogan-Finnemore M : Delivering 21st century Antarctic and Southern Ocean science. *Antarct Sci.* 2016;28(6):407–423. 10.1017/S0954102016000481

[ref-24] Kloser RJ Ryan TE Keith G : Deep-scattering layer, gas-bladder density, and size estimates using a two-frequency acoustic and optical probe. *ICES J Mar Sci.* 2016;73(8):2037–2048. 10.1093/icesjms/fsv257

[ref-70] Leray M Yang JY Meyer CP : A new versatile primer set targeting a short fragment of the mitochondrial COI region for metabarcoding metazoan diversity: Application for characterizing coral reef fish gut contents. *Front Zool.* 2013;10(1): 34. 10.1186/1742-9994-10-34 23767809 PMC3686579

[ref-25] Mackie GO Pugh PR Purcell JE : Siphonophore biology.In: *Adv Mar Biol*. Elsevier,1988;24:97–262. 10.1016/S0065-2881(08)60074-7

[ref-26] Mapstone GM : Global diversity and review of Siphonophorae (Cnidaria: Hydrozoa). *PLoS One.* 2014;9(2): e87737. 10.1371/journal.pone.0087737 24516560 PMC3916360

[ref-27] Munro C Vue Z Behringer RR : Morphology and development of the Portuguese man of war, *Physalia physalis*. *Sci Rep.* 2019;9(1): 15522. 10.1038/s41598-019-51842-1 31664071 PMC6820529

[ref-28] Oksanen J Blanchet FG Friendly M : vegan: Community Ecology Package. R package version 2.64. In.2022. Reference Source

[ref-29] Ortman BD Bucklin A Pages F : DNA barcoding the Medusozoa using mtCOI. *Deep Sea Res Pt II: Top Stud Oceanogr.* 2010;57(24–26):2148–2156. 10.1016/j.dsr2.2010.09.017

[ref-30] Park N Choi H Shin KH : Distribution of siphonophores in the Northwest Pacific Ocean and links to environmental conditions. *Front Mar Sci.* 2023;10: 1223477. 10.3389/fmars.2023.1223477

[ref-31] Parry H Atkinson A Somerfield P : A metabarcoding comparison of taxonomic richness and composition between the water column and the benthic boundary layer. *ICES J Mar Sci.* 2021;78(9):3333–3341. 10.1093/icesjms/fsaa228

[ref-32] Pontin D Cruickshank R : Molecular phylogenetics of the genus *Physalia* (Cnidaria: Siphonophora) in New Zealand coastal waters reveals cryptic diversity. *Hydrobiologia.* 2012;686(1):91–105. 10.1007/s10750-011-0994-8

[ref-33] Proud R Handegard NO Kloser RJ : From siphonophores to deep scattering layers: uncertainty ranges for the estimation of global mesopelagic fish biomass. *ICES J Mar Sci.* 2019;76(3):718–733. 10.1093/icesjms/fsy037

[ref-34] Robison BH : Deep pelagic biology. *J Exp Mar Biol Ecol.* 2004;300(1–2):253–272. 10.1016/j.jembe.2004.01.012

[ref-35] Robison BH Reisenbichler KR Sherlock RE : Seasonal abundance of the siphonophore, *Nanomia bijuga,* in Monterey Bay. *Deep Sea Res Pt II: Top Stud Oceanogr.* 1998;45(8–9):1741–1751. 10.1016/S0967-0645(98)80015-5

[ref-36] Schwartz MK Luikart G Waples RS : Genetic monitoring as a promising tool for conservation and management. *Trends Ecol Evol.* 2007;22(1):25–33. 10.1016/j.tree.2006.08.009 16962204

[ref-37] Sommer SA Van Woudenberg L Lenz PH : Vertical gradients in species richness and community composition across the twilight zone in the North Pacific Subtropical Gyre. *Mol Ecol.* 2017;26(21):6136–6156. 10.1111/mec.14286 28792641

[ref-38] Sunagawa S Acinas SG Bork P : *Tara* Oceans: towards global ocean ecosystems biology. *Nat Rev Microbiol.* 2020;18(8):428–445. 10.1038/s41579-020-0364-5 32398798

[ref-39] Suter L Polanowski AM Clarke LJ : Capturing open ocean biodiversity: comparing environmental DNA metabarcoding to the continuous plankton recorder. *Mol Ecol.* 2021;30(13):3140–3157. 10.1111/mec.15587 32767849

[ref-40] Turon X Antich A Palacín C : From metabarcoding to metaphylogeography: separating the wheat from the chaff. *Ecol Appl.* 2020;30(2): e02036. 10.1002/eap.2036 31709684 PMC7078904

[ref-41] Uribe-Palomino J López R Gibbons MJ : Siphonophores from surface waters of the Colombian Pacific Ocean. *J Mar Biol Ass UK.* 2019;99(1):67–80. 10.1017/S0025315417002065

[ref-42] Warren J Stanton T Benfield M : *In situ* measurements of acoustic target strengths of gas-bearing siphonophores. *ICES J Mar Sci.* 2001;58(4):740–749. 10.1006/jmsc.2001.1047

[ref-43] Weigand H Beermann AJ Čiampor F : DNA barcode reference libraries for the monitoring of aquatic biota in Europe: gap-analysis and recommendations for future work. *Sci Total Env.* 2019;678:499–524. 10.1016/j.scitotenv.2019.04.247 31077928

[ref-71] WoRMS Editorial Board: World Register of Marine Species.2023; Accessed 2023-11-01. 10.14284/170

