# Supplementary material for: Global distribution patterns of siphonophores across horizontal and vertical oceanic gradients
Source: Open Res Eur. 2024 Aug 13;4:177. [Version 1] doi: 10.12688/openreseurope.18226.1 (PMC11399771; doi:10.12688/openreseurope.18226.1)
Supplement: Peer review history

# Reviewer report: Reviewer response for version 1

**DOI:** 10.21956/openreseurope.19696.r43203

**Published:** 2024-09-13

**Authors:** Iole Di Capua

**Affiliations:** 1 Stazione Zoologica Anton Dohrn Napoli, Naples, Campania, Italy

Review of the manuscript: Global distribution patterns of siphonophores across horizontal and vertical oceanic gradients by C. Claver and co-authors.

 The authors have been making concerted efforts to document the biological diversity and the distribution of a key component taxon of planktonic communities at a global scale using the COI gene, and now, many of these data will be available for the scientific community. The potential to use DNA metabarcoding for gelatinous organisms, also with a complex colonial structure is one the most important part of this manuscript, especially due to a sad decrease in expert taxonomists and the few molecular data are already available on this hydrozoan species, and most of them worldwide distributed and also present in the deep-layers, due to a very peculiar biological cycle.

 If my concerns can be addressed successfully in a minor revision, and the paper should be indexed.

 The few aspects are:

 • Please specify during the taxonomic assignments the percentage of similarity of OTUs used for the analyses, considering that the assignation of the siphonophores is at the species level.

 • Please, include an additional table with standard sample information, such as the type of multi-net uses for zooplankton collection, the mesh size of the net, the filtered water volume by zooplankton nets etc.

 Moreover, please note that recently, using the same gene in two Mediterranean sites (Di Capua et al., 2024), many hydrozoans, including seven siphonophores species, have been detected, mainly in the dipper layer (200-0m) in a submarine canyon.

Is the study design appropriate and does the work have academic merit?

Yes

Is the work clearly and accurately presented and does it cite the current literature?

Yes

If applicable, is the statistical analysis and its interpretation appropriate?

Yes

Are all the source data underlying the results available to ensure full reproducibility?

Yes

Are the conclusions drawn adequately supported by the results?

Yes

Are sufficient details of methods and analysis provided to allow replication by others?

Partly

Reviewer Expertise:

Zooplankton integrative taxonomy and ecology

I confirm that I have read this submission and believe that I have an appropriate level of expertise to confirm that it is of an acceptable scientific standard.

## References

[1] : Integrative approach to monitoring metazoan diversity and distribution in two Mediterranean coastal sites through morphology and organismal eDNA. *Sci Rep* .2024;14(1) : 10.1038/s41598-024-69520-2 19291 10.1038/s41598-024-69520-2 39164301 PMC11336219

# Reviewer report: Reviewer response for version 1

**DOI:** 10.21956/openreseurope.19696.r43197

**Published:** 2024-09-11

**Authors:** Ana Baricevic

**Affiliations:** 1 Center for Marine Research, Ruder Boskovic Institute, Rovinj, Croatia

The research enabled a detailed and extensive description of the diversity and distribution of one of the poorly described groups of zooplankton. The results are clearly presented and based on clear and accurate methods of data collection and processing. The discussion is constructive and the conclusions are correctly drawn. I believe that the authors could only state better in the results and emphasize which of the species found are newly recorded for each individual oceanic area.

Is the study design appropriate and does the work have academic merit?

Yes

Is the work clearly and accurately presented and does it cite the current literature?

Yes

If applicable, is the statistical analysis and its interpretation appropriate?

Yes

Are all the source data underlying the results available to ensure full reproducibility?

Yes

Are the conclusions drawn adequately supported by the results?

Yes

Are sufficient details of methods and analysis provided to allow replication by others?

Yes

Reviewer Expertise:

Marine plankton diversity. Molecular methods (omics) for plankton community characterisation.

I confirm that I have read this submission and believe that I have an appropriate level of expertise to confirm that it is of an acceptable scientific standard.
